# Have farmers had enough of experts?

**DOI:** 10.1007/s00267-021-01546-y

**Published:** 2021-10-11

**Authors:** Niki A. Rust, Petra Stankovics, Rebecca M. Jarvis, Zara Morris-Trainor, Jasper R. de Vries, Julie Ingram, Jane Mills, Jenny A. Glikman, Joy Parkinson, Zoltan Toth, Regina Hansda, Rob McMorran, Jayne Glass, Mark S. Reed

**Affiliations:** 1 Centre for Rural Economy, School of Natural and Environmental Sciences, Newcastle University, Kings Road, Newcastle, NE1 7RU UK; 2 Department for Environment, Food and Rural Affairs, Lancaster House, Hampshire Court, Newcastle-Upon-Tyne, NE4 7YH UK; 3 Institute of Advanced Studies Kőszeg, Chernel u. 14, Kőszeg, 9730 Hungary; 4 Georgikon Campus, Institute of Agronomy, Hungarian University of Agriculture and Life Sciences, 16 Deák Ferenc Str, 8360 Keszthely, Hungary; 5 School of Science, Auckland University of Technology, 55 Wellesley Street East, Auckland CBD, Auckland, 1010 Aotearoa New Zealand; 6 Sustainable Fibre Alliance, 84 Seatown, Cullen, Buckie, AB56 4SL UK; 7 Strategic Communication Group, Wageningen University, Hollandseweg 1, 6706 KN Wageningen, The Netherlands; 8 Countryside and Community Research Institute (CCRI), University of Gloucestershire, Francis Close Hall, Cheltenham, GL50 4AZ UK; 9 Instituto de Estudios Sociales Avanzados (IESA-CSIC), Campo Santo de los Mártires 7, 14004 Córdoba, Spain; 10 Griffith Business School, Griffith University, 170 Kessels Rd, Nathan, QLD 4111 Australia; 11 Georgikon Campus, Institute of Agronomy, Hungarian University of Agriculture and Life Sciences, 16 Deák Ferenc Str, Keszthely, 8360 Hungary; 12 Thriving Natural Capital Challenge Centre and Rural Policy Centre, Department of Rural Economy, Environment & Society, Scotland’s Rural College (SRUC), Peter Wilson Building, Kings Buildings, West Mains Road, Edinburgh, EH9 3JG UK

**Keywords:** Innovation, Social learning, Social media, Sustainable agriculture, Technology adoption, Trust, Soil management

## Abstract

The exponential rise of information available means we can now, in theory, access knowledge on almost any question we ask. However, as the amount of unverified information increases, so too does the challenge in deciding which information to trust. Farmers, when learning about agricultural innovations, have historically relied on in-person advice from traditional ‘experts’, such as agricultural advisers, to inform farm management. As more farmers go online for information, it is not clear whether they are now using digital information to corroborate in-person advice from traditional ‘experts’, or if they are foregoing ‘expert’ advice in preference for peer-generated information. To fill this knowledge gap, we sought to understand how farmers in two contrasting European countries (Hungary and the UK) learnt about sustainable soil innovations and who influenced them to innovate. Through interviews with 82 respondents, we found farmers in both countries regularly used online sources to access soil information; some were prompted to change their soil management by farmer social media ‘influencers’. However, online information and interactions were not usually the main factor influencing farmers to change their practices. Farmers placed most trust in other farmers to learn about new soil practices and were less trusting of traditional ‘experts’, particularly agricultural researchers from academic and government institutions, who they believed were not empathetic towards farmers’ needs. We suggest that some farmers may indeed have had enough of traditional ‘experts’, instead relying more on their own peer networks to learn and innovate. We discuss ways to improve trustworthy knowledge exchange between agricultural stakeholders to increase uptake of sustainable soil management practices, while acknowledging the value of peer influence and online interactions for innovation and trust building.

## Introduction

We are now living in a world experiencing more information overload than ever before (Bawden and Robinson 2020). Never has there been a time when people can theoretically, with the right technological resources and skills, access much of humanity’s collective knowledge in a matter of seconds via the internet. This explosion of digital content has, however, led to numerous challenges. Firstly, there is now such an abundance of content that locating the information needed can be difficult (Holton and Chyi 2012). Secondly, the rise of inaccurate, unchecked, unintentionally or deliberately misleading and pre-filtered content can make it harder to know what is trustworthy, credible and reliable (Hargittai et al. 2010). Together, this can create anxiety, uncertainty, or irrationality in decision-making (Eppler and Mengis 2004), creating a significant cognitive load, which can lead to inefficient and ineffective use of time and resources (Bawden and Robinson 2020). Information seekers are therefore not only challenged with finding information but also assessing, processing and controlling it (Tenopir 1990; Jackson and Farzaneh 2012).

To overcome the problem of making sense of voluminous and conflicting information, we tend to use simple heuristics, or mental short-cuts, to assess information (Hmielowski et al. 2014). Trust is a heuristic used to evaluate information and is based on numerous factors, such as: whether the new information appears compatible with what is already believed, if it comes from a credible source and if it appears to be believed and trusted by peers (Lewandowsky et al. 2012). Traditionally, ‘experts’, who are thought to contain a high degree of detailed knowledge and expertise on a particular topic (such as scientists and doctors) have been considered trustworthy sources of information. Academic researchers are commonly thought of as ‘experts’ in their field of study, and studies have found widespread public trust in both science and scientists across 140 countries (Wellcome Trust 2019). In a 2020 analysis across six countries and 80,000 respondents, the Reuters Institute found scientists and doctors were the most trusted source of information on coronavirus—ranking higher than national and global health organisations, and well above governments and the media (Newman et al. 2020). However, these studies focused on *generalised* trust but did not look at trust towards *specific* scientists. This nuance is important because people may trust science or scientists in general, but not science related to particular topics or scientists who represent them (Nadelson et al. 2014). For instance, though people in the UK generally claim to trust scientists, the level of trust towards government scientists regarding information on genetically modified (GM) food has historically been low (Grove White et al. 1997; Marris et al. 2001; Seifert 2020; Ipsos MORI 2021; Busch et al. 2021). Similarly, controversies surrounding bovine spongiform encephalopathy (mad cow disease) during the 1990s prompted public criticism of the role of scientific advice in policy-making, leading to the formulation of rules for science-based policy-making by the Government. This suggests the extent to which someone trusts a particular expert on a specific topic is often related to whether that ‘expert’ appears to hold similar values and interests to them (Siegrist and Cvetkovich 2000; Earle 2010).

Given soil degradation on farms is a global problem (Lal and Stewart 1990), there have been calls for changes in farming soil management practices to be more sustainable (Rust et al. 2020). However, uptake of more sustainable soil management practices has remained low (Alskaf et al. 2020; Lahmar 2010). This is concerning, given soil is the foundation upon which most of our food is produced but is created so slowly that it could be considered non-renewable on a human timescale (Lal 2015). Intensive agricultural practices degrade soil thus there is a dire need to increase the adoption of products and practices that conserve soil (Thomas et al. 2018). Indeed, the need for sustainable soil management techniques is included in Sustainable Development Goal 15, noting its importance for healthy soils to help to create more sustainable futures. Trust in information about soil management has been noted as a key factor influencing uptake of more sustainable practices (Rust et al. 2020). Farmer motivations to implement more sustainable agricultural practices are complex and include agronomic, economic, environmental, political, psychological and social factors (Baumgart-Getz et al. 2012; Prager and Posthumus 2010; Ulrich-Schad et al. 2017; Dessart et al. 2019). When determining a farmer’s likelihood of adopting more sustainable management approaches and technologies, it is crucial that farmers have access to good quality information from sources perceived as trustworthy (Mills et al. 2017; Joffre et al. 2020).

Trust in information is also related to risk: it can be risky to change behaviour, especially if outcomes are uncertain, so acting on information where risks are high depends partly on the amount someone trusts the information (Ilbery et al. 2013; Slovic 2000; Millstone and Van Zwanenberg 2000; Dandy 2012). For farmers, some changes in farm management may carry a high risk to their livelihood, especially if the change is substantial, costs are high or the payoff not instant; here, farmers tend not to trust information that comes from people with limited farming experience (Mauro et al. 2009; Rust et al. 2020; Skaalsveen et al. 2020). Moreover, consumer attitudes may change far faster than new practices can be developed (bearing in mind that there can be time lags of over 20 years in many cases between agricultural research and ultimate adoption of innovations; Alston 2008). For instance, previous studies found that the riskiness of changing practices was a key barrier stopping farmers from using soil conservation measures in Australia and the US (Pannell et al. 2006; Arbuckle and Roesch-McNally 2015). Costs are high, there is frequently no price premium to be gained and consumer attitudes change far faster than farmers are able to keep pace. Alston et al. report ag R&D lags of over 20 years, suggesting that on farm ROI lags would extend beyond the short term of 3–5 years.

Knowing which sources of soil information that farmers—and the people who advise them—use and trust, would be of great value for advocates of sustainable agriculture, as this information can be used to understand who may be the best messengers for sharing information on sustainable agricultural practices. Historical studies highlighted the agricultural adviser as a valued, trusted and respected source of information and mediator of sustainable agricultural practice adoption for farmers (Angell et al. 1997; Fearne 1991), albeit with often complex relationships evolving (Ingram 2008). However, as it has become more challenging for farmers to navigate through the complex and often messy network of pluralistic advisory services (Klerkx and Proctor 2013), farmers have increasingly turned to their farmer peers rather than traditional ‘experts’ such as agricultural advisers (Wood et al. 2014; Skaalsveen et al. 2020; McMorran 2021), especially small-scale farmers, who often struggle to pay for independent advisory services (Sutherland et al. 2017). The advent of the internet, coupled with a growth of digital content, presents an information-seeking challenge for farmers who must decide which sources of information on soil to use and trust. Whilst it has been noted that some farmers are now using the internet to learn about farm management in general (Defra 2019), it is not yet known whether they are also using online sources to learn specifically about new soil management practices (Mills et al. 2019). Similarly, with the rise in disinformation and fake news (Martens et al. 2018), we do not know how farmers are now validating information nor which sources of information encourage farmers to implement more sustainable soil management practices. Moreover, ‘cognitive dissonance’ may arise from apparently contradictory research, on the one hand urging a shift from the post World War II paradigm of food security using input-intensive agriculture (Lang and Barling 2012) towards sustainable agriculture (Donkers 2014); and on the other hand recognising that organic agriculture is unlikely to provide long term food security (Badgley and Perfecto 2007; Connor 2018).

In this study, we address these research gaps using a case study of farmers in Hungary and the UK—two countries experiencing growing levels of soil degradation (Environment Agency 2019; Szilassi et al. 2006). To more thoroughly understand the journey that farmers as information seekers take when learning about and implementing new soil management approaches, this study was guided by the following research questions:

1. Which sources of soil information do farmers and those who advise farmers use and trust?
2. How is this information validated?
3. Who influences farmers to implement new soil management practices?

## Material and Methods

We took an inductive, qualitative approach to this research using a case study design (Merriam 1998), allowing us to gain an in-depth understanding of the topic (O’leary 2004). We drew upon persuasion theories and diffusion of innovations theory to situate our study conceptually. Persuasion theories posit that someone is more likely to change their behaviour if the beliefs that underpin their attitudes change (Petty et al. 1991; Okumah et al. 2020). This process is affected by the information shared, its source and the characteristics of the information receiver (Blackstock et al. 2010), as well as how this information conveys social norms e.g. which agricultural practices are normally used and how they are applied in practice (Cialdini et al. 2006). Persuasion theories stipulate that trusted sources are thought to be more effective vehicles of change compared with untrusted sources (Petty et al. 1991). To complement this, the theory on diffusion of innovation posits that external and internal factors influence an individual’s decision to innovate; external factors include characteristics about the innovation itself alongside ‘change agents’ (also known as influencers), plus the social network and communication channels (Rogers 2003).

We undertook semi-structured interviews during 2019 with UK farmers and advisers and at the start of 2020 for Hungarian farmers and advisers. UK advisors tended to operate from the private sector while Hungarian advisors were more likely to come from the public sector. We recognise that, increasingly, there is overlap between these stakeholders, such as where some farmers are also advisers or have advanced degrees. However, due to limited space in this paper, we categorise stakeholders based on the group in which the respondent primarily categorised themselves. We chose these two case studies as contrasting examples: the UK being a cooler climate and being a long-standing member of the EU (although the research was conducted just after the country voted to leave the European Union) and Hungary being a warm semi-continental climate joining the EU in 2004 having transitioned from communism. Their agricultural knowledge and innovation systems (AKIS) had historically been distinctly different, though have more recently become similarly pluralistic (Ingram and Mills 2019). In Hungary, due in part to the transition from communism to capitalism, state-run farms that had an important role in AKIS were replaced by fragmented public and private organisations (Nemes and High 2013; Biró 2017). Hungary now has a complex advisory network, including free, partly subsidised and privatised AKIS institutions. Like Hungary, the AKIS network in the UK has become increasingly pluralistic over the last number of decades (Knierim et al. 2017), with paid-for advice primarily coming from advisors working for large agribusinesses or independent consultants, though some state-funded AKIS also exist, through focused programmes such as the Catchment-Based Approach to water management.

An interview guide was developed with input from the authors, informed by the literature and the theoretical concepts described above, and questions were trialled on a subset of the target populations. Following feedback from these pilots, questions were adapted to improve the clarity of question wording. The study obtained ethical approval by the Newcastle University Ethics Committee. All respondents were asked for their free, prior informed consent to take part in the research and accepted verbally. We used a purposive sampling strategy to source respondents based on the judgement of the researchers (Etikan et al. 2016) by targeting soil advisers via online agricultural databases, contacting regional farmer groups and attending agricultural meetings, supplemented by snowball sampling, where farmers recommended their colleagues for interview. Farmers in our sample included arable and mixed farmers located in south-western Hungary and across the UK. We did not sample livestock-only farmers as soil quality is a more significant issue on arable farms (Environment Agency 2019). A total of 43 farmers and 39 advisers were interviewed across the two countries (Table 1). Just over half (45/82) of the interviews took place over the phone, with the remainder taking place in person. All interviews were recorded with consent from the respondents and lasted on average 48 min (with a range of 24–106 min).

**Table 1:** Characteristics of interview respondents

Country	Profession	Gender
Hungary	*Adviser: (11)*	F (1), M (10)
	Agribusiness (6)	
	Farming association (1)	
	Independent (2)	
	University/college (2)	
	*Farmer: (11)*	F (4), M (7)
	Arable (10)	
	Mixed (1)	
UK	*Adviser: (28)*	F (13), M (15)
	Agribusiness (6)	
	Agricultural levy board (5)	
	Environmental NGO (3)	
	Farming association (2)	
	Government (2)	
	Independent (5)	
	University/college (5)	
	*Farmer: (32)*	F (5), M (27)
	Arable (13)	
	Mixed (19)	

New respondents were sourced until theoretical saturation had been reached, where no new themes were emerging from the data (O’leary 2004). The recordings were transcribed (and first translated into English if they were conducted in Hungarian) then analysed in QSR NVivo^®^ (QSR International, version 12). Coding was undertaken by the first author inductively using thematic analysis, which involved first reading the transcripts to acquire an overview of the interview data and then coding each interview based on common themes emerging from the data. The results are presented as summaries of the parent themes that emerged related to our research questions; anonymised quotes have been included where they provide examples of these themes.

## Results

**Fig. 1:**
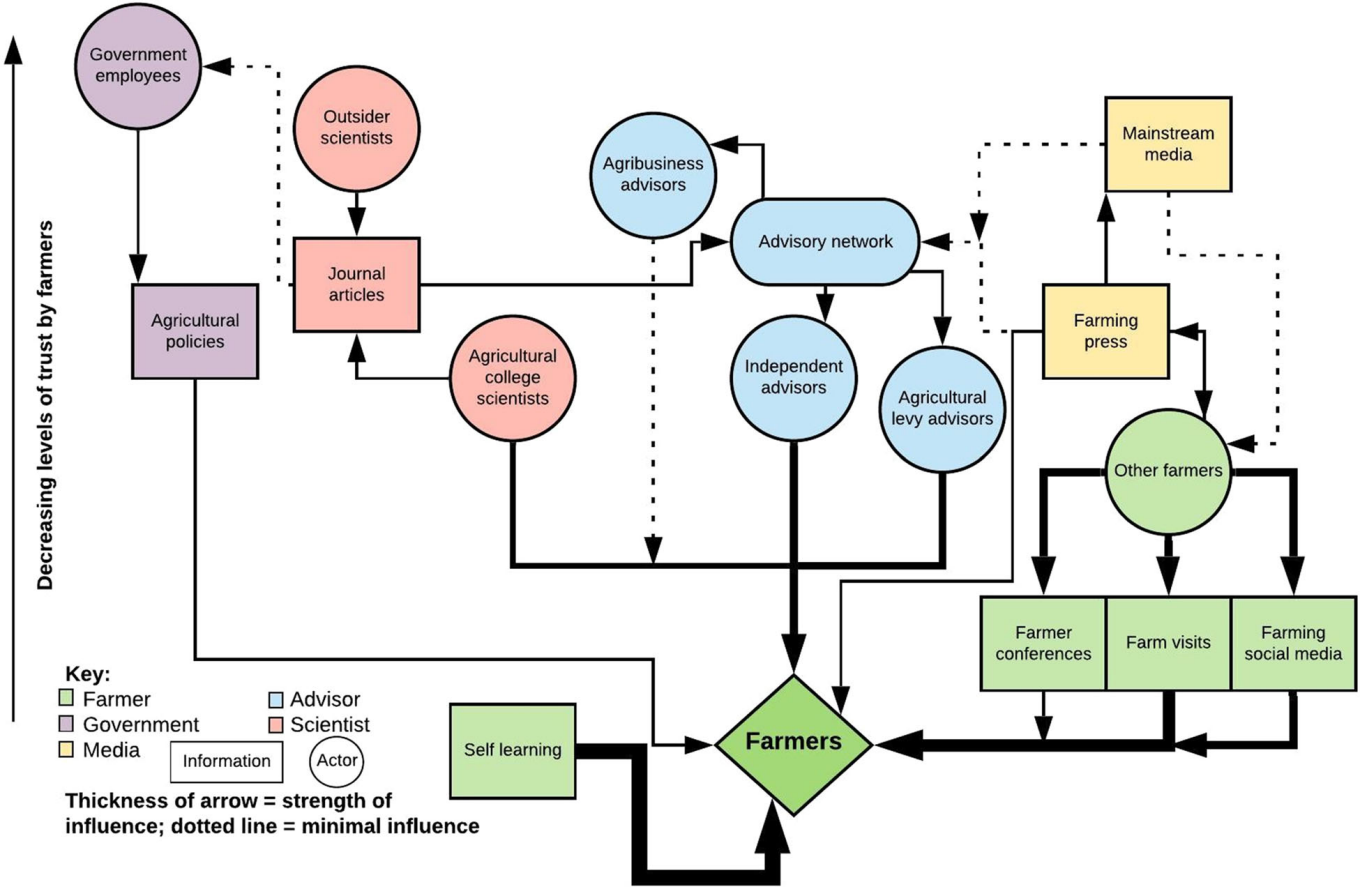
A conceptual map of the information sources used and trusted by Hungarian and UK farmer respondents, as well as who influenced these farmers to implement a new soil management practice

Results are summarised in Fig. 1, showing the sources used and trusted, as well as who influenced farmers to implement new soil management practices. These are then described in greater detail in the subsequent sections, considering which sources of soil information farmers and advisors use and trust, how this information is validated, and who influences farmers to implement new soil management practices.

### Information Sources and Trust

Farmers and advisers in Hungary and the UK used diverse sources to gain new information about soil, ranging from individuals (such as other farmers) to institutions (such as agricultural levy boards^1^). Farmers generally used and placed most trust in other farmers when they wanted to learn about soil management, as one Hungarian arable farmer (HF07) explained:

“I discuss our experiences with my fellow farmers. We are in continuous contact; we keep in touch but if some problem occurs then we can just call each other like: “What did you do? How do you deal with it? What are you results? How big is the yield? What is the quality?”. We can trust in each other’s experiences because we are friends, so we do not want to cause trouble to each other, and we know how the others works; we see the result on each other farms with our eyes.”

Respondents also frequently used digital technology to find information: for farmers, this was usually via social media and other online sources such as farming forums and the agricultural press; for advisers, this was primarily in online scientific journals. Most respondents thought the internet was a useful way to find out about new information on soil and gauge what other farmers felt about the topic, though advisers (particularly those from colleges/universities) did not find online farmer experiences shared on social media were credible as they believed anecdotal stories were not robust enough to be considered “evidence”. Farmers, however, reported that they valued social media to learn directly from and connect with other farmers about agricultural practices and experiences much easier than they could in person. Both Hungarian and British farmers used social media as a source of agricultural information, though this practice tended to be more common in British farmers. One British advisor commented:

“Twitter has become a very important tool for farmers to learn peer to peer… Farmers sharing just real-life case studies. They are sharing their trials; they just share what they are doing and you see farmers interact with that. Twitter has played a central role in providing one form of evidence that farmers do trust which is what other farmers are doing. Twitter has empowered that to happen.”

Whether talking about other farmers in the social media or geographical networks, farmers tended to get information from other farmers with similar farming experiences and interests to them, rather than seeking out particularly experienced or knowledgeable farmers, and when they sought evidence beyond these networks, they were typically cautious. Whilst farmers and advisers regularly used the internet to gather soil information, many were somewhat cautious of fully trusting online sources, as one British mixed farmer (BF04) explained: “*Ultimately the internet is the biggest tool of useless information but also the biggest tool of useful information. It is just knowing what bit of information you are reading at the time*.” Therefore, the internet was considered useful to gather information but that this information needed to be taken “*with a pinch of salt*”—as numerous respondents mentioned—before it could be believed, trusted and acted upon.

Both advisers and farmers used the farming press to gather information on soil, with farmers placing more trust in this source than advisers. However, both farmers and advisers reported a degree of scepticism in the content of the farming press, believing the content to be somewhat swayed by the agribusinesses who pay for adverts in the magazines. For example, one UK advisor commented, “*If you read Farmer’s Weekly you think ‘really?’… You read most of it and there is an underlying message in it, there is always a commercial message*.” National general press sources such as broadsheet newspapers were not trusted as sources of information on soil as these were deemed to have a different agenda to the agricultural sector.

Farmers and advisers alike mentioned that established agricultural levy/membership organisations were trusted sources of information about soil because respondents believed these organisations undertook reliable, robust science that was practical for on-farm use. Here, respondents trusted the research produced from these institutions as the institutions themselves were perceived as trustworthy and being on the side of the farmer. For example, one UK farmer and advisor commented, *“I would probably as a general rule tend to be fairly trusting of anything which comes out of AHDB* [Agriculture and Horticulture Development Board] *probably from the point of view that again it is independent, it is levy funded and certainly in the last year or two they seem to have swung into a fairly pragmatic attitude of looking at things both from a scientific and economic point of view and they tend to bring in good researchers.”* Respondents also felt these institutions were empathetic towards what farmers wanted and communicated in ways farmers understood. Farmers often mentioned that partly why they trusted agricultural levy boards was because they were funded by the farming industry.

Whilst farmers trusted the research produced from these agricultural levy boards (though less than they trusted other farmers), they generally only trusted academic research institutions—such as agricultural colleges—that were known for longstanding work on agriculture conducted in collaboration with farmers. Researchers from other institutions, such as governments, non-agriculturally focused colleges and universities (hereafter denoted as “outsider scientists”), were not trusted by farmers, as these researchers were thought to have a conflicting agenda to farmers so did not have farmers’ best interests at heart. In Hungary, this was partly due to the changing advisory landscape, as a university adviser explained (HA10):

“Unfortunately, the world has changed a lot. State-financing universities are no longer the case. Information is difficult to pass on. Farmers are less and less likely to believe in university research.”

Therefore, the degree to which farmers trusted scientific outputs was dependent on the institutions producing them. Numerous farmers in Hungary and the UK were suspicious of the funding sources that outsider scientists relied on, assuming that many research projects were part-funded by (what they deemed) untrustworthy and biased donors such as the agrochemical industry; this was one reason why most farmers placed little trust in advisers who worked for large agribusinesses.

The way in which outsider scientists obtained funding was seen to “*corrupt*” their results, as a British mixed farmer (BF27) proposed. Other farmers mentioned an additional reason for not using information from or trusting outsider scientists was because they could not access academic research, partly as it was often behind paywalls and partly because it was written in a way that farmers found hard to understand. Outsider scientists were also thought to work at different geographic scales to farmers, as the researchers often wanted large-scale generalisable studies whereas farmers wanted locally specific research applicable to their farm. This fed into the suspicion farmers had towards outsider scientists, as the knowledge produced by these researchers was not generally actionable on the farm and was communicated in ways farmers struggled to understand, suggesting a lack of empathy that outsider scientists conveyed towards farmers. The scalar difference was thought to affect the usefulness of scientists’ advice, as reflected upon by a British agricultural college adviser (BA23):

“Farmers quite rightly trust other farmers and why would they trust the scientists, the scientist who only writes a paper? And why would they trust a scientist who doesn’t turn up in their field and talk to them directly? Why would they read a paper and think ‘I must try that out’?”

### Agricultural Information Validation

Agricultural information on soil was often validated by respondents using a rough triangulation process, including collecting additional evidence (like repeated trials or studies done in different contexts) and getting verification from trusted sources, as this Hungarian agribusiness adviser (HA08) explained:

“I do not trust without any doubt, so I do cross analyses and if I find out that more papers state the same then I trust in the information provided.”

There were concerns about the rise of “fake news”, with farmers likely to triangulate potentially unreliable sources with other farmers. For example, one British farmer said:

“I think in the last few years there has been more of fake news but the things I read I don’t think are fake. I don’t know actually. Gut feeling maybe, talking to other farmers. I wouldn’t just jump in with both feet and do something regardless. You talk to other farmers and see what they are doing”. Advisors, on the other hand, were more likely to triangulate with other published sources. One British advisor checked her sources by “understanding where the information has come from, who is putting it out and whether they have any prior experience”.

However, there was a difference in the sources and methods respondents chose to verify information. Advisers were more likely to look for perceived scientific robustness, as this Hungarian agribusiness adviser (HA01) described: *“I like university research. It is verified back statistically and biologically, confirmed results and a reliable source.”* Conversely, farmers often relied on other farmers to verify information, as a British farmer (BF01) explained: “*Farmers want to see other farmers doing it… it convinced you more when you actually see it and see what the benefits are, that is the crucial thing*”. Demonstration farms and visits were important for some respondents, for example one UK advisor commented, *“demonstration events [are a] particularly really powerful way to have a look at it. I think particularly with soil demonstrations that until you see it, it is very hard to visualise it and see what works and doesn’t work.”* This verification process was used to overcome the challenges with information overload and misleading information, as a British agribusiness adviser (BA15) described:

“I built up a network of what I call stakeholders that I will go to and test in terms of their opinion on certain products or certain techniques and what their view is.”

Scientific advisers rarely needed in-person verification and were content with trusting journal articles, especially those from purported reputable sources deemed to be those that had higher impact factors and written from authors they knew. For farmers, however, they needed tangible proof from a similar farming context, as a Hungarian farmer (HF01) explained:

“Show me a fellow farmer who tried that practice and it worked on his field. It is also important that it should be emphasised that it worked in the same kind of natural environment like my farm. It has produced results under similar circumstances than mine.”

Farmers also tended to rely on sensory verification by seeing, touching, even smelling the soil to determine how a new management practice was affecting the soil. In effect, sensing was believing.

### Implementation of Innovative Soil Management Practice Influencers

Farmers interviewed in both Hungary and the UK frequently mentioned they were motivated to try new soil management practices for financial reasons, either to save money or due to government subsidies/fines. However, in instances where risks of changing practices and/or the investment costs were high, farmers were influenced to implement more sustainable soil practices by recommendations from trusted peers, primarily other farmers. Whilst farmers interviewed often preferred in-person proof, some did not necessarily need to have met trusted peers in person, as one British farmer (BF32) described when talking about why he decided to try more sustainable soil management practices:

“I am quite taken with trust in [two prominent agricultural advisers], what they are saying… Those people, what they are saying is correct and it is not going to cost you a lot to try what they are doing, to compost or to improve your soil, they are not multi-nationals so they are not trying to sell you anything.”

This farmer explained the reason why he trusted these individuals was because he felt they understood farmers and provided useful advice, again conveying the importance of empathy that information providers offered to information receivers. This farmer mentioned he had learnt of these people from the internet and consumed their digital content to find out more about soil. A few other farmers (both in Hungary and the UK) also said they got inspiration to try a new soil management practice by social media posts from farmers they followed online. However, for the majority of farmers, it was not just one person or piece of information that prompted them to change, but a process of building up knowledge from various sources before acting, as a Hungarian farmer (HF04) recalled her reasons for trying composting: “*I have heard about it from my mother-in-law first, then I started to look at it on the internet*”.

Whilst the internet was a useful resource for most farmers to learn about soil (including in the verification process), it was not usually the main factor influencing farmers to change their practices. Many farmers interviewed were motivated based on seeing farms be successful with the practice. Whilst some British farmers interviewed did, to some degree, trust the advice from their agricultural advisers, information from other farmers had a stronger influence and tended to be preferred and acted upon more than information from advisers, as one Hungarian agribusiness adviser (HA08) noted: “*In Hungary, professional advisers do not count so much for the farmers. In many cases, if the neighbour starts spraying the chemical, so will he*.”. Similarly, a British farmer commented, *“It is a great thing to have a neighbour and you see what he is doing and you can see some of his good crops and some of his bad bits… Neighbours that talk and banter is a great thing to progress.”*

This suggests that, ultimately, social influences affecting uptake were strong, and it was mostly other farmers who prompted farmers to make changes to their soil management.

## Discussion

Information sharing has changed with the growth of online sources, therefore this research examined the sources of information farmers now use and trust for soil management. Farmer influencers are increasingly important for the dissemination of agricultural information. They are not just celebrities; they can be farmer influencers who share credible and trustworthy information which are important for the adoption of sustainable farming innovations (Rust et al. 2020). Furthermore, the growth of social media platforms has introduced a whole new breed of relatable, and easily accessible, farmer influencers (Zhang et al. 2020). For example, this role was found to be significant for the learning and decision-making of no-till farmers in UK (Skaalsveen et al. 2020). Working with these types of influencers as part of a communication strategy may help governments reach farming audiences in a way that feels more relatable than traditional information dissemination methods including “experts”. Instead of *talking to* the audience using experts, employing farmer influencers can open up new communication channels, creating an easily accessible information source that *communicates with* the modern farmer.

Unsurprisingly, in contrast to previous studies (Chowdhury and Odame 2013; Kaushik et al. 2018; Mills et al., 2018), farmers and advisers in both Hungary and the UK are now frequently using digital channels to seek information on soil as it is freely accessible and useful, with farmers using agricultural social media sites and advisers regularly using online journals. Whilst most advisers in this study were sceptical of the soil management claims on social media sites, farmers were more willing to consume social media content from respected farmer “influencers”. Farmers are increasingly drawing on social media farmer influencers (Zhang et al. 2020) for information and these farmer influencers are the online version of Rogers’ (2003) opinion leaders or champions, who have the ability to inflence the diffusion of innovations. Influencers are important as they share endorsed opinions on social media platforms, which can help disseminate information quickly and broadly, and change norms about behaviours and practices (Kay et al. 2020). These farmer influencers also provide tangible evidence of the benefits of new management practices and technologies on farm, reducing the perceived risks associated with change (McKitterick et al. 2019). Consistent with studies examining the role of social networks on farming practices (Skaalsveen et al. 2020), findings from this study show a shift away from traditional sources of information such as broadsheet newspapers and periodicals towards digital and interpersonal sources including farmer influencers, which are often perceived as more credible and trustworthy.

The findings from this study provide support for Phillipov and Goodman’s (2017) argument that farmers are increasingly becoming celebrities in the same vein as celebrity chefs, and have the ability to influence the food system as a whole. Indeed, farmer networks on Twitter have been found to be quite strong and dense, with farmers tending to group with other farmers, with research scientists and other advisers on the periphery (Meador et al. 2021). These close-knit farmer social networks may enhance exchange of soil knowledge and uptake of innovations through growth of in-group social capital (Rust et al. 2020). This resonates with the finding of Mills et al. (2018) that virtual communities of practice are developing around soil management on Twitter in which farmer champions are emerging that are highly respected by other farmers. However, there are risks with only interacting with people who share similar views and beliefs through curating your own social media bubble (Colleoni et al. 2014). These risks include susceptibility to confirmation bias (Nickerson 1998) and content bias (Tian and Chao 2012), through seeking information that matches pre-existing beliefs (Colleoni et al. 2014). Such an approach could exacerbate lack of trust between “insiders” and perceived “outsiders”, even when the information of “outsiders” could provide additional benefits to farmers. Using trusted, perceived similar intermediaries may help to expand knowledge exchange beyond social media bubbles, building more bridging social capital to increase the chance of innovation (Rust et al. 2020). Opportunities also exist for “expert” advisers and others working in agricultural and rural sectors to engage on Twitter with well-connected individuals who can act as ‘brokers’ between stakeholder networks and “launch” information between science, policy and farmers (Meador et al. 2021).

In contrast to historic agricultural research (e.g. Eldon 1988; Fearne 1991), we found that it was not necessarily the traditional “expert” advisers that farmers preferred to go to when they wanted to learn about soil—instead, they often chose to learn from other farmers. In contrast to North America, where agronomists and agri-business companies are often viewed as trusted sources (e.g. Fransoo 2018), farmers in our research were less trusting of these sources. This may be in part due to the more recent privatisation of advice services in Europe, compared to North America where these actors have played a more prominent role in the advice system for longer. It may also in part be due to a wariness of biased commercial advice or in some cases limited expertise in soil management (Ingram 2008). That farmers are now turning to their peers for advice and support has also been observed in more recent studies (Joffre et al. 2020; McKitterick et al. 2019; McMorran 2021) as other farmers are perceived to not have a conflict of interests, and instead have applied, practical experience that is more relevant to the innovating farmer (Inman et al. 2018). Tsouvalis et al. (2000) argued that farmers particularly value knowledge gained through farming experience, and in turn value researchers and advisors who work directly with farmers. Using blended learning approaches have also been found to be important, for example, in addition to online sources, farmers also valued learning from experiences such as visiting sites where the new practices were being successfully implemented by other farmers (Cullen et al. 2016). This is consistent with experiential learning as argued for by Tsouvalis et al. (2000) and is particularly important for soil management (Ingram 2010). For example, Stoate et al. (2019) studied five participatory research projects working with farmers, concluding that direct engagement with farmers builds trust within the farming community, resulting in a greater shared understanding of how to address their soil management objectives. Farmers in our study were more trusting of other farmers and more likely to change their soil management based on farmers’ recommendations, indicating that social learning (Reed et al. 2010) through trusted, similar peers—such as other farmers—is important for farmers to be persuaded to act on that information. Applying the Diffusion of Innovation theory (Rogers, 1995), farmers raise their own awareness of soil issues via their social network, which leads to evaluation (i.e. verification) and then on to trials. In effect, farmers see themselves as the experts (Palmer et al. 2009).

Having said this, farmers in our study also often trusted agricultural levy boards and researchers at agricultural colleges due to their longstanding relationship with farmers, which allowed these institutions to build up credibility over time (Sutherland et al. 2013). It was important that information providers were perceived to have the farmers’ best interests at heart—a factor shown to increase trust (Head 2012). This is consistent with the experiential learning where farmers value information from researchers or technology developers who work with farmers (Stoate et al. 2019). Levy boards were believed to have this empathetic trait as these institutions were paid for by farmers and were thought to have shared values. Empathy and social similarity towards the farmer were therefore key in building trust (Neef and Neubert 2011).

With the growth of the Internet and social media, in contemporary society people have become more vocal about mistrust or a lack of confidence in information from some sources. When it came to distrusted sources, farmers from both countries did not trust “outsider scientists” (such as those not from agricultural levy boards or agricultural colleges). This is consistent with previous research where fabricated media stories—such as biased research produced by research institutions partnering with agrichemical industries (Blakemore 2018)—can undermine farmers’ perceptions of the research community (Stroud 2018). The recent farmer protests in India, one of the largest and longest acts of resistance against newly constituted farm laws, is a classic example of farmer anxieties and breakdown of trust between the government and farmers (Narayanan 2020; Mohan and Mistry 2020). The core problem was the inadequate stakeholder consultation before formulating laws, and inadequate discussion in parliament before laws were passed; and the perception amongst farmers that the laws were made in keeping with the interests of corporates in agribusiness, instead of the farmers (Mohan and Mistry 2020). Multiple rounds of talks between the government and the farm union leaders failed, and the debate continues to play out politically, through the media and social media, where distrust has been expressed in noted agriculture economists whose think pieces have been labelled as biased.

In addition to a perceived lack of empathy, farmers in our study did not trust outsider scientists due to the way information was communicated. To increase trust farmers need information that is accessible and easy to understand rather than advice provided being too technical to understand (Halabi and Carroll 2015). Building farmers’ trust in scientific recommendations for sustainable soil management technologies may, in addition to using a trusted, perceived similar third party, require careful translation of academese into communication styles more applicable for different farmer groups (Clark and Murdoch 1997). Another reason for the distrust by farmers from both countries towards scientists was reportedly because scientists had different goals to farmers: outsider scientists were thought to want journal article publications and research funding rather than create direct benefits for farmers. In effect, there was a perceived lack of homophily or perceived similarity between farmers and outsider scientists, supporting the use of diverse networks (Klerkx and Proctor 2013) and particularly farmers’ social networks (Skaalsveen et al. 2020).

Those interviewed in this research emphasised differences between farmers and certain types of trusted advisor, versus researchers and those (including certain advisors) with commercial interests. Farmers contested traditional notions of scientific “experts” versus those with “lay” or “local” knowledge, reframing farmers as the experts, based on experiential, situated knowledges and skills that were rooted in practical experience. The emphasis was on trust rather than expertise, exploring the various reasons why certain information sources were considered to be trustworthy or not, reframing expertise as the provision of information or knowledge that could be trusted. This resonates with calls to exercise caution over the use of terms such as expert, lay or local in relation to knowledge (Wynne 1996), which tends to emphasise difference and keep different knowledge communities apart. Instead, Tsouvalis et al. (2020) write about “knowledge cultures” to emphasise the complex processes through which knowledge is formed and transformed through social interaction and power relations, and how these processes in turn shape social norms, behaviours and cultures, as farmers derive meaning from the knowledge they embody through their farming practice.

## Conclusions

We have found that as more farmers get online, they are building digital relationships with other farmers to form communities of practice. Farmers from Hungary and the UK were found to be using farming social media as a key source of agricultural information and some were motivated to change their farming practices based on information received from digital farmer influencers and validated by their peers. As more agribusinesses become digital-first, the power of the internet—including social media—is likely to continue to grow, shaping our lives in ways previously unimaginable. Whilst our study found social media to play a more minor role in influencing the majority of farmers interviewed to change their farming practices, it is possible that, over time, its influence may grow, and farmers may find new experts upon which to rely. Future research should expand sample sized and employ social network analysis to identify the important influencers within the farmers ‘network (Del Fresno García et al. 2016) and understand why farmer influencers appear to be a credible source (Lewandowsky et al. 2012); conducting this for online farmer communities of practice would be useful to help pinpoint the most promising influencers who are already trusted in farmer networks. Meador et al (2021) have employed such an approach in Scotland and this signalled the significant potential for social network analysis to assess how government and academic institutions can better identify and engage with online influencers to disseminate information, increase impact and encourage uptake of new agricultural practice. Whether via social network analysis or a purposive sample for future qualitative research, it would be useful to identify and interview the early adopters who were trusted by the farmers in our sample, most of which appeared to be mid- to late-adopters. Furthermore, all the farmers in our sample had adopted some sort of sustainable soil innovation that they were able to talk about, and future research might seek to identify non-adopters for interview to understand factors influencing their decisions.

Identifying farmer influencers will be critical for disseminating reliable and trustworthy agricultural information to ensure and accelerate the adoption of sustainable farming innovations. Social media users as information seekers perceive influencers as trusted and experienced voices of authority on specific topics, so these influencers are useful messengers to share information. Working with known and trusted third parties, such as these online influencers, early on and throughout the knowledge exchange process could help promoters of sustainable soil management practices share information more effectively with farmers (Breetz et al. 2005; Hansen 2002), which we suggest may result in greater uptake. An influencer with a farming background is likely to be more effective at influencing other farmers (Rust et al. 2020), as trust is partly based on the experience and occupation of the person sharing information (Blackstock et al. 2010). However, drawing from Tsouvalis et al. (2020), it is also important to recognise that farmer influencers might increase the credibility of information, but for the actual uptake of soil improving techniques, it is critical to encourage place-based, ‘new knowledge cultures’, which are backed by adequate, long-term institutional support. This is where some insights can be drawn from some of the global South countries (e.g. Indonesia, Uganda, India) which have addressed issues of trust and behavioural change around sustainability and food security by forming farmer field schools (FFS). At its core, the FFS is an innovative pedagogical approach which emphasises ecological learning, systems analysis, and experimentation for and by groups of farmers who meet routinely for field-based sessions during an entire production cycle to learn how to make adaptive soil and crop management decisions, and find local solutions (Mfitumukiza et al. 2020; FAO 2019; Charatsari et al. 2020). An adapted version, in the form of ‘farmers’ knowledge networks’ or something similar could be considered at the landscape level to enable farmers to adapt or alter their agricultural practices to changing circumstances and concerns around soil heath. These new knowledge cultures could engage farmers to become their own researchers, observers and decision-makers (action research/ Innovations model), rather than expecting them to follow the standard, linear technology transfer model, which invariably is top-down, with little room to accommodate farmers’ own agency and interests in decision-making. de Bruyn et al. (2017) argue that to address the issue of distrust and reliability between key stakeholders, there is a need for a more creative approach to engage with farmers. A dynamic learning environment, in this case virtual and/or on ground, which maintains farmers’ interests, and is based on trust and mutual respect of each other’s knowledge and perspectives could be the way forward.

Despite moving away from traditional ‘experts’ and a growing reliance on farmer-farmer knowledge exchange networks, there may be a more important role for trusted intermediaries than ever before. These knowledge brokers can enhance the exchange of ideas between groups, who might not naturally gain trust directly via homogenous in-groups. The knowledge brokers’ ability to connect different groups could also be embraced to expand more insular networks or individuals, both with sustainable soil practices and other beneficial practices. This may be particularly important to enable farmers and researchers to learn about innovations arising from each other’s work.

## Footnotes

1 Levy boards, such as the Agricultural and Horticultural Development Board in the UK, charge a statutory levy to farmers, growers and others in the supply chain to fund research and development and provide advice to the sector.
